# The validity and reliability of the Slovenian version of the health literacy questionnaire short-form (HLS-EU-Q16) among adults and older adults

**DOI:** 10.3389/fpubh.2024.1474539

**Published:** 2024-12-11

**Authors:** Monika Lamot, Andrej Kirbiš

**Affiliations:** Department of Sociology, University of Maribor, Maribor, Slovenia

**Keywords:** HLS-EU-Q16, health literacy, Slovenia, adults, older adults, validity, reliability

## Abstract

**Introduction:**

Health literacy is an important predictor of health behavior and self-rated health, playing a crucial role in shaping public health outcomes. Valid and reliable health literacy assessments are essential for effectively tailoring health interventions, particularly in different cultural contexts. Several questionnaires have been developed to measure health literacy, including the widely used 47-item Health Literacy Questionnaire and its shorter versions. However, the validity of these shorter and more time efficient HLS-EU versions, such as the 16-item HLS-EU-Q16, has been less extensively researched in different age subgroups. Given Slovenia’s aging population and the differences in health literacy between adults and older adults, it is important to assess whether the HLS-EU-Q16 performs reliably across these groups. Therefore, the present study aimed to examine the validity and reliability of the 16-item health literacy questionnaire (HLS-EU-Q16) in Slovenia across two age groups: adults and older adults.

**Methods:**

We analyzed representative Slovenian sample data from the Health Literacy of Adults study. The sample included 2,327 adults aged 23–64 years (53.7% women) and 876 older adults aged 65–99 years (55.7% women).

**Results:**

Construct validity revealed a modified three-factor structure of the HLS-EU-Q16 among adults, reflecting a broader conceptualization of health literacy that encompasses overlapping domains of health promotion, disease prevention, and health care. In contrast, a two-factor structure emerged among older adults, indicating a more consolidated approach where these domains are perceived as part of an integrated health management strategy. This suggests that adults may conceptualize health literacy across multiple, yet interconnected dimensions, while older adults approach it more holistically. Convergent validity, reliability, concurrent-predictive, concurrent, and discriminant validity results were satisfactory for both age groups. Predictive validity, however, provided somewhat inadequate results, as the tool poorly predicted certain health behaviors, such as smoking, alcohol consumption, and physical activity, in both age groups.

**Conclusion:**

Overall, the HLS-EU-Q16 demonstrated adequate validity and reliability among both adults and older adults, indicating that it is an appropriate instrument for assessing health literacy in Slovenia.

## 1 Introduction

Health literacy, defined as the ability to understand and use information to make health decisions (1), is measured using various scales and questionnaires. One prominent tool is the Health Literacy Questionnaire (HLS-EU), originally consisting of 47 items across three dimensions and domains: healthcare, disease prevention, and health promotion, which include accessing, understanding, evaluating, and using health information (2). Due to its length, shorter versions with 16 items (2), 12 items (3), and 6 items (4) were developed. This study focuses on the validity of the HLS-EU-Q16 in Slovenia, a tool whose satisfactory psychometric properties have been confirmed in India (5), Portugal (6), and Greece (7). However, the HLS-EU-Q16 has not been extensively validated for different age sub-populations, particularly in older adults. Although older adults have been included in validation studies as part of the general population (8, 9), they have not been examined separately.

Specific tools for assessing health literacy among older adults have been developed. For example, the Rapid Estimate of Inadequate Health Literacy (REIHL) (10) and the TLSA questionnaire in Taiwan (11) both show favorable psychometric properties. Given the widespread use of the HLS-EU-16 (12–14), it is crucial to validate it in the Slovenian context, taking into account cultural factors that may affect its validity. Health literacy is a dynamic construct shaped not only by cognitive abilities but also by life experiences, social opportunities, and the broader cultural and societal context (15). As highlighted by Lorini et al. (15), healthcare, education, social systems, cultural norms and media sources, can influence how health literacy manifests across different populations. In Slovenia, factors such as healthcare access, educational background, and social norms may affect how individuals engage with and understand health information. Moreover, since older adults exhibit lower health literacy levels (16), lower functional literacy (17), and issues with health information in media (18), examining the validity and reliability of the HLS-EU-Q16 specifically for this population is essential.

Lower health literacy has been shown to directly impact health outcomes and healthcare utilization. For instance, Cho et al. (19) found that improving health literacy in older adults could enhance health outcomes and reduce the use of costly hospital and emergency services. Furthermore, Mirzaei et al. (20) demonstrated that health literacy levels significantly affect the effectiveness of educational interventions, especially in improving nutrition knowledge and behaviors among the older adult, with higher health literacy leading to greater improvements.

Studies assessing the validity of the HLS-EU-Q16 often focus on construct validity, neglecting discriminant and predictive validity (9, 14, 21, 22). Additionally, some studies report internal reliability as a single factor without analyzing the factorial structure (6, 15). Previous studies often identify multiple factors within the health literacy construct, but subsequent analyses, like correlations or regressions, often treat it as a single factor without confirming a second-order structure (12, 13). The aim of the present research is to present a more comprehensive analysis of the validity and reliability of the questionnaire separately for the two age groups.

### 1.1 Psychometric structure of the HLS-EU-Q16 questionnaire

The three-dimensional factorial structure of the HLS-EU-Q16, following the theoretical framework, has been confirmed in adults aged 25–65 years (5). CFA (Confirmatory Factor Analysis) indicated a good fit for the three-dimensional model, which includes healthcare, disease prevention, and health promotion. High correlations between these dimensions suggest they are closely related and collectively represent the broader construct of health literacy. In this study, older adults were included alongside adults (21). This finding aligns with an Exploratory Factor Analysis (EFA) study that found a unidimensional structure, suggesting the HLS-EU-Q16 can function as a one-factor questionnaire with strong psychometric properties and reliability (23). Notably, Mousum et al. (14) confirmed both the three-factor and one-factor solutions in a population that included both adults and older adults, indicating that the questionnaire can function as either a single-factor or a three-factor scale.

*H1*: The HLS-EU-Q16 demonstrates a three-factor solution among Slovenian adults.

*H2*: The HLS-EU-Q16 demonstrates a single-factor solution among Slovenian adults.

Eronen et al. (18) examined individuals aged 66 and above in Finland, reporting good test–retest reliability of the HLS-EU-Q16 but without addressing other indicators of reliability or validity. Another study involving individuals aged 72–92 years found poor reliability of HLS-EU-Q16 (24). Furthermore, differential item functioning (DIF) analyses revealed age-related differences in HLS-EU-Q16 responses. Specifically, older adults found it more difficult to understand what their doctor says and to judge when to seek a second opinion compared to younger individuals. These findings suggest that certain items in the HLS-EU-Q16 may not be equally valid across different age groups. However, CFA for age subgroups was not reported, leaving the construct validity for different age groups unclear (4). Another study also did not report the factorial structure for age subgroups but identified age-related differences in items such as finding information on treatments and judging when to seek a second opinion, which were more challenging for older adults (25). Consequently, the factorial structure of the HLS-EU-Q16 among older adults remains unknown.

*RQ1*: What is the factorial structure of the HLS-EU-Q16 among Slovenian older adults?

### 1.2 Discriminant validity

Discriminant validity of health literacy questionnaires has been assessed using various methods in previous studies. For instance, Elsworth et al. (26) and Maindal et al. (27) examined discriminant validity of the Health Literacy Questionnaire (HLQ) by analyzing inter-factor correlations and factor loadings within factors. Abacigil et al. (28) assessed the discriminant validity of the HLS-EU-Q47 by comparing participant scores across different age groups, educational levels, genders, health statuses, and socioeconomic statuses, ensuring the questionnaire could distinguish effectively between these groups. Similarly, Sun et al. (29) evaluated discriminant validity of the Oral Health Literacy Scale by comparing scores between the highest and lowest scoring groups, finding significant differences that confirmed the scale’s ability to distinguish effectively between these groups.

Discriminant validity can also be evaluated in relation to other distinct constructs. Pelikan et al. (3) analyzed the discriminant validity of the 12-item HLS19-Q12, a shorter version of the HLS-EU-Q47, by correlating it with digital, communicative, and navigational health literacy measures, which are related but distinct constructs. Their study demonstrated good discriminant validity for the 12-item questionnaire in comparison to these other constructs. Previous studies have not assessed the discriminant validity of the HLS-EU-Q16 questionnaire by comparing it with communicative and navigational health literacy measures. Therefore, in this study, we focus on the discriminant validity of the HLS-EU-Q16 in relation to communicative and navigational health literacy. Communicative health literacy pertains to the skills required for active participation in interactions with healthcare providers, including expressing treatment preferences and asking clarifying questions (30). Navigational health literacy refers to the ability to locate, comprehend, and use information to effectively navigate the healthcare system, such as accessing services and making informed healthcare decisions (31). By adopting this approach, we aim to evaluate whether the HLS-EU-Q16 can effectively distinguish between these theoretically distinct constructs of health literacy.

*H3*: The HLS-EU-Q16 demonstrates discriminant validity in relation to communicative and navigational health literacy questionnaires among adults (H3a) and older adults (H3b).

### 1.3 Concurrent-predictive validity

Concurrent predictive validity was assessed based on the approach used by Pelikan et al. (3), who examined sociodemographic and socioeconomic factors as determinants of health literacy. Cross-country studies, such as Sørensen et al. (16), have demonstrated that individuals with lower socioeconomic status, lower educational attainment, and older age tend to exhibit lower health literacy. Similarly, research from Australia confirmed that individuals with lower levels of education experienced greater difficulty understanding and accessing health information. Furthermore, women scored slightly lower than men on some dimensions of the health literacy scale (32). In Spain, lower health literacy scores were observed among individuals over 65, those with incomplete secondary education, and those who were unemployed, across several dimensions of health literacy (33). These findings support the relevance of sociodemographic and socioeconomic factors in establishing the concurrent-predictive validity of health literacy measures.

### 1.4 Predictive validity

Predictive validity is often examined by checking whether health literacy predicts various health outcomes, since health literacy is known as a predictor of subjective health (34) and health behaviors (35, 36). Suka et al. (37), for example, found that those exhibiting higher health literacy were less likely to smoke, drink or lack exercise. Predictive validity of the HLS-EU-Q16 has previously been confirmed for predicting poorer self-assessment of health, higher prevalence of disability, obesity, lower physical activity, and less frequent consumption of fruits and vegetables (38). The same holds true for older adults, as those with lower health literacy are more likely to engage in unhealthy behaviors such as smoking, alcohol consumption, and lower physical activity (39). Additionally, we assessed predictive validity while controlling for sociodemographic and socioeconomic determinants, which are significant predictors of health behavior. For instance, smoking is more prevalent among men, individuals with lower education, less household wealth, those living in rural areas, and older adults (40). Moreover, higher income levels are associated with higher fruit intake (41), while younger individuals and men have higher alcohol consumption (42).

*H4*: When controlling for sociodemographic and socioeconomic confounders, the HLS-EU-Q16 statistically significantly predicts better self-rated health (H4a), less frequent smoking (H4b), alcohol consumption (H4c), more frequent physical activity (H4d) and healthy eating (H4e).

## 2 Materials and methods

### 2.1 Sample

We analyzed data from the Health Literacy of Adults in Slovenia (HLS-SI19) (43), involving 3,360 participants, 53.7% of whom were women. Participants ranged from 18 to 99 years old (M = 51.6). The data was collected as part of a larger European study M-POHL. The Slovenian data were collected through a combination of computer-assisted personal interviews (CAPI), self-administered questionnaires (SAQ), and computer-assisted web-based interviews (CAWI). Participants were selected using a multi-stage random sampling method. The inclusion criteria required all participants to be permanent residents aged 18 and above. Respondents were informed that they could opt out of the survey at any time (43). As the present study aimed to validate the HLS-EU-Q16 questionnaire in two groups—adults and the older adult—the characteristics of each subgroup are presented below.

#### 2.1.1 Adults

The adult group comprised 2,327 participants aged 23–64 years (M = 45.2 years), with 53.4% women. Of the participants, 65.4% were employed, 12.9% retired, 8.4% unemployed, and 7.2% self-employed. Additionally, 2.1% were students, 1.8% homemakers, and 1.3% unable to work. The average self-assessed socioeconomic status was mid-level (M = 5.49; SD = 1.59). Educational attainment was diverse: 25.3% reported completing secondary vocational education, 16.2% lower or intermediate vocational education, 14.4% second-degree higher education, 11.6% general secondary education, 9.8% higher vocational education, 8.7% first-degree higher education, and 3.8% had a master’s or doctorate.

#### 2.1.2 Older adults

The older adults group included 876 participants aged 65 to 99 years (M = 73.9 years), with 55.7% women. Most were retired (96.3%), with a small percentage still working. The average self-assessed socioeconomic status was slightly below average (M = 5.18; SD = 1.56). The majority of the sample reported having completed secondary vocational education (24.4%), followed by primary education (24.0%), lower or intermediate vocational education (23.4%), higher vocational or college education (8.3%), second-degree higher education (5.4%), first-degree higher education (2.1%), and 0.7% had a master’s or doctorate.

### 2.2 Measures

#### 2.2.1 Health literacy questionnaire

The Slovenian version of the HLS-EU-Q47 questionnaire was used solely to verify concurrent validity of the HLS-EU-Q16. The primary focus of the research was to validate HLS-EU-Q16, which comprises 16 items across three domains: healthcare, disease prevention, and health promotion (6). Participants responded using a four-point scale (1 = very difficult; 4 = very easy). An example item includes “How easy or difficult is it for you to find information on treatments of illness that concern you” (see Table 1 for the full list).

**Table 1:** Explorative factor analysis on the adult population.

Item	Factor 1	Factor 2	Factor 3
Find information on treatments of illness that concern you.			0.62
Find out where to get professional help when you are ill?			0.67
Understand what your doctor says to you?			0.77
Understand your doctor’s or pharmacist’s instruction on how to take a prescribed medicine.			0.59
Judge when you may need to get a second opinion from another doctor.			0.60
Use information the doctor gives you to make decisions about your illness.			0.70
Follow instructions from your doctor or pharmacist.			0.47
Find information on how to manage mental health problems like stress or depression.	0.50		
Understand health warnings about behavior such as smoking, low physical activity and drinking too much.	0.76		
Understand why you need health screenings?	0.60		
Judge if the information on health risks in the media is reliable.		0.72	
Decide how you can protect yourself from illness based on information in the media.		0.76	
Find out about activities that are good for your mental well-being.	0.77		
Understand advice on health from family members or friends.	0.58		
Understand information in the media on how to get healthier.		0.57	
Judge which everyday behavior is related to your health.	0.73		

Extraction method: Principal Component Analysis. Rotation method: oblique with Kaiser normalization. Rotation converged in 12 iterations. KMO = 0.941. Bartlett’s test of sphericity was statistically significant [χ^2^(120) = 14034.085, *p* < 0.001].

#### 2.2.2 Communicative health literacy

Communicative health literacy was measured with 11 statements on a four-point scale (1 = very difficult; 4 = very easy). Example questions included: “How easy or difficult is it to describe to your doctor the reasons for your visit?,” “Explain your health concerns to your doctor?,” and “Understand the words your doctor uses?.” The scale demonstrated excellent internal reliability (Cronbach’s *α* adults = 0.94, Cronbach’s α older adults = 0.95).

#### 2.2.3 Navigational health literacy

Navigational health literacy was assessed with 12 statements, such as, “Understand information about how the healthcare system works (e.g., what types of healthcare services are available),” “Judge which type of healthcare service you need in case of a health problem,” and “Judge how much of the healthcare service is covered by your health insurance (e.g., whether co-payments are required).” Participants responded on a four-point scale (1 = very difficult; 4 = very easy). The scale also showed excellent internal reliability (Cronbach’s *α* adults = 0.93, Cronbach’s α older adults = 0.94).

#### 2.2.4 Self-rated health and health behaviors

Subjective health was assessed with a standard self-rated health question using a five-point scale (1 = very good; 5 = very poor). Health behaviors examined included frequency of smoking, alcohol consumption, physical activity, and fruit and vegetable intake. Participants reported their weekly frequency of these behaviors (99 = never, 0 = less than once a week, 1 = one day, 2 = two days, 3 = three days, 4 = four days, 5 = five days, 6 = six days, 7 = seven days). Variables were recoded from 0 (never) to 8 (7 days).

#### 2.2.5 Sociodemographic and socioeconomic determinants

The analysis also included sociodemographic and socioeconomic variables, including gender (1 = male, 2 = female), education (1 = no formal education; 11 = master’s degree, doctorate), self-assessed socioeconomic status (1 = lowest socioeconomic level in society; 10 = highest socioeconomic level in society), and self-assessed economic deprivation, measured with the question “How easily can you pay your living expenses each month?” (1 = very easily; 4 = very difficult).

### 2.3 Statistical analyses

This study evaluated the validity and reliability of the shorter version of the health literacy questionnaire, HLS-EU-16, using Mplus 8.3 and SPSS 29. We examined construct, convergent, discriminant, concurrent-predictive, and predictive validity. Construct validity was assessed using confirmatory factor analysis (CFA), with the following model fit indices: chi-square (χ^2^), comparative fit index (CFI > 0.90), Tucker-Lewis index (TLI > 0.90), root mean square error of approximation (RMSEA <0.08), and standardized root mean square residual (SRMR <0.08) (44). For construct validity, explorative factor analysis (EFA) was also employed. Convergent validity was evaluated through standardized factor loadings, average variance extracted (AVE > 0.50), and composite reliability (CR > 0.70) as recommended by Fornell and Larcker (45). High AVE and CR values indicate good convergent validity, meaning the indicators adequately explain the latent constructs. Internal reliability was assessed using Cronbach’s alpha (*α* > 0.70), indicating the degree of internal consistency of the measurement instrument (46). Furthermore, discriminant validity was assessed following the methodology of Pelikan et al. (3), using the navigational and communicative health literacy questionnaires. We used correlation analysis (Pearson’s coefficient) to determine whether the constructs were distinct from each other. A range of correlation coefficients between 0.40 and 0.70 was considered indicative of good discriminant validity. Concurrent-predictive validity was examined with linear regression where we employed the same predictors as Pelikan et al. (3), namely sociodemographic and socioeconomic predictors. Next, concurrent validity was verified by correlating the HLS-EU-Q47 questionnaire with the shorter HLS-EU-Q16 questionnaire. A high correlation (*r* > 0.75) indicates high concurrent validity (47). Predictive validity was assessed using sociodemographic and socioeconomic factors, health behaviors, and self-rated health as predictors. The inclusion of health literacy (HLS-EU-Q16) in the model was examined using hierarchical regression to determine if health literacy contributed to additional explained variance in health behaviors and self-rated health (45).

## 3 Results

### 3.1 Construct validity

We first assessed the construct validity of the questionnaire using confirmatory factor analysis (CFA). CFA was performed on the questionnaire’s three theoretically proposed dimensions (factors): health care, disease prevention, and health promotion (results shown in Supplementary Table S1). Based on the model fit statistic, we conclude that the theoretically proposed three-factor structure of the HLS-EU-Q16 questionnaire falls below the recommended thresholds for model fit among Slovenian adults and older adults, indicating its use may not be appropriate. In accordance with Hypothesis 2, we also examined the single-factor structure of the questionnaire in both age groups (Supplementary Table S2). The analyses revealed that the usage of the one-factor HLS-EU-Q16 among adults and older adults is also not appropriate, as the model fit statistics fell below the recommended thresholds.

Due to the unsatisfactory model fit, we conducted an exploratory factor analysis (EFA) to examine the factor structure of the questionnaire in both age groups (Tables 1, 2). In the adult population, the analysis largely confirmed a three-dimensional factor structure for the HLS-EU-Q16 questionnaire, but the dimensions were not identical to the original structure (Table 1). Items related to finding and understanding health information and doctors’ instructions (health care) primarily loaded onto Factor 3. Items related to disease prevention loaded onto Factor 2. Items related to health promotion mainly loaded onto Factor 1. However, some health care and disease prevention items, such as “find out about activities that are good for your mental well-being” and “understand advice on health from family members or friends” also loaded onto Factor 1. This suggests that adults may interpret health promotion more broadly, incorporating aspects of disease prevention and health care into their perception of health enhancement.

**Table 2:** Explorative factor analysis on the older adult population.

Item	Factor 1	Factor 2
Find information on treatments of illness that concern you.	0.53	
Find out where to get professional help when you are ill?	0.76	
Understand what your doctor says to you?	0.82	
Understand your doctor’s or pharmacist’s instruction on how to take a prescribed medicine.	0.90	
Judge when you may need to get a second opinion from another doctor.	0.42	
Use information the doctor gives you to make decisions about your illness	0.68	
Follow instructions from your doctor or pharmacist.	0.80	
Find information on how to manage mental health problems like stress or depression.	0.50	
Understand health warnings about behavior such as smoking, low physical activity and drinking too much.	0.81	
Understand why you need health screenings?	0.75	
Judge if the information on health risks in the media is reliable.		0.83
Decide how you can protect yourself from illness based on information in the media.		0.86
Find out about activities that are good for your mental well-being.	0.67	
Understand advice on health from family members or friends.	0.47	
Understand information in the media on how to get healthier.		0.64
Judge which everyday behavior is related to your health.	0.69	

Extraction method: Principal Component Analysis. Rotation method: oblique with Kaiser normalization. Rotation converged in 5 iterations. KMO = 0.949. Bartlett’s test of sphericity was statistically significant [χ^2^(120) = 6642.715, *p* < 0.001].

A two-factor solution of the questionnaire emerged in the older adult population (Table 2), which indicated a different structure from the original HLS-EU-Q16 structure. Specifically, items related to health care and health promotion primarily loaded onto the first factor, suggesting older adults perceive these aspects as more interconnected in comparison to the originally proposed factor structure of the questionnaire. Items related to disease prevention, along with some health promotion items about media reliability and protective measures, loaded onto the second factor. This indicates that older adults may integrate health care, disease prevention, and health promotion into a more cohesive concept, consolidating items into fewer factors compared to the original HLS-EU-Q16 structure.

After the exploratory factor analysis revealed the factor structure of the questionnaire in both populations, we further confirmed it with CFA (Table 3). The fit indices were consistent with recommended values, indicating that the questionnaire structure identified with EFA is suitable for both age groups.

**Table 3:** Construct validity of the HLS-EU-Q16 Questionnaire.

	Adult population	Older adult population
χ^2^(*df*)	1245.075 (101)***	739.387 (103)***
CFI	0.92	0.91
TLI	0.91	0.90
RMSEA	0.07	0.08
SRMR	0.04	0.05

CFI, Comparative Fit Index; TLI, Tucker-Lewis Index; RMSEA, Root Mean Square Error of Approximation; SRMR, Standardized Root Mean Square Residual. ***p < 0.001.

Next, standardized factor loadings among the adult population (Table 4) showed satisfactory values, exceeding the recommended threshold for all three factors. Factor 1 loadings ranged from 0.50 to 0.70, Factor 2 loadings ranged from 0.62 to 0.71, and Factor 3 loadings ranged from 0.67 to 0.75. All factor loadings were statistically significant at *p* < 0.001. Regarding average variance extracted (AVE), the evidence for convergent validity was less satisfactory. AVE values for Factors 1, 2, and 3 were 0.43, 0.46, and 0.49, respectively, all below the recommended threshold, with Factor 3 being the closest. On the other hand, composite reliability (CR) demonstrated better convergent validity, with values of 0.84 for Factors 1 and 2, and 0.74 for Factor 3, all above the 0.70 threshold (results not presented in a table).

**Table 4:** Standardized factor loading in the adult population.

Factor	Item	Factor loading	S.E.	Est./S.E.	*p*
Factor 1	Find information on treatments of illness that concern you	0.63	0.01	44.826	0.000
	Find out where to get professional help when you are ill	0.70	0.01	56.183	0.000
	Understand what your doctor says to you	0.68	0.01	52.142	0.000
	Understand your doctor’s or pharmacist’s instruction on how to take a prescribed medicine	0.70	0.01	56.014	0.000
	Judge when you may need to get a second opinion from another doctor	0.50	0.02	28.520	0.000
	Use information the doctor gives you to make decisions about your illness	0.67	0.01	50.007	0.000
	Follow instructions from your doctor or pharmacist	0.68	0.01	52.339	0.000
Factor 2	Find information on how to manage mental health problems like stress or depression	0.70	0.01	56.553	0.000
	Understand health warnings about behavior such as smoking, low physical activity and drinking too much	0.71	0.01	59.781	0.000
	Understand why you need health screenings	0.65	0.01	47.543	0.000
	Find out about activities that are good for your mental well-being	0.71	0.01	59.402	0.000
	Understand advice on health from family members or friends	0.62	0.02	42.296	0.000
	Judge which everyday behavior is related to your health	0.69	0.01	53.813	0.000
Factor 3	Judge if the information on health risks in the media is reliable	0.67	0.02	42.470	0.000
	Decide how you can protect yourself from illness based on information in the media	0.67	0.02	43.172	0.000
	Understand information in the media on how to get healthier	0.75	0.01	52.627	0.000

S.E., Standard error; Est./S.E., Estimate divided by Standard Error.

For the older adult sample, the questionnaire also demonstrated good convergent validity based on standardized factor loadings (Table 5). Factor 1 loadings ranged from 0.58 to 0.78, and Factor 2 loadings ranged from 0.71 to 0.76, with all indicators being statistically significant at *p* < 0.001. The good convergent validity was further supported by the average variance extracted (0.48 for Factor 1 and 0.54 for Factor 2) and composite reliability results, which were 0.92 for Factor 1 and 0.78 for Factor 2.

**Table 5:** Standardized factor loading in the older adult population.

Factor	Item	Factor loading	S.E.	Est./S.E.	*p*
Factor 1	Find information on treatments of illness that concern you	0.60	0.02	26.129	0.000
	Find out where to get professional help when you are ill	0.68	0.02	34.766	0.000
	Understand what your doctor says to you	0.67	0.02	33.963	0.000
	Understand your doctor’s or pharmacist’s instruction on how to take a prescribed medicine	0.78	0.02	52.068	0.000
	Judge when you may need to get a second opinion from another doctor	0.58	0.02	24.429	0.000
	Use information the doctor gives you to make decisions about your illness	0.69	0.02	36.659	0.000
	Follow instructions from your doctor or pharmacist	0.73	0.02	42.351	0.000
	Find information on how to manage mental health problems like stress or depression	0.70	0.02	36.957	0.000
	Understand health warnings about behavior such as smoking, low physical activity and drinking too much	0.78	0.02	53.638	0.000
	Understand why you need health screenings	0.74	0.02	44.498	0.000
	Find out about activities that are good for your mental well-being	0.76	0.02	47.984	0.000
	Understand advice on health from family members or friends	0.61	0.02	26.975	0.000
	Judge which everyday behavior is related to your health	0.69	0.02	35.592	0.000
Factor 2	Judge if the information on health risks in the media is reliable	0.71	0.02	31.664	0.000
	Decide how you can protect yourself from illness based on information in the media	0.74	0.02	34.399	0.000
	Understand information in the media on how to get healthier	0.76	0.02	36.999	0.000

S.E., Standard error; Est./S.E., Estimate divided by Standard Error.

We further assessed the internal consistency or reliability of the HLS-EU-16 questionnaire. For the adult group, all three dimensions of the HLS-EU-Q16 demonstrated good reliability. Specifically, Cronbach’s alpha was 0.83 for Factor 1 (mainly *health promotion* and some *health care* items), 0.84 for Factor 2 (*disease prevention*), and 0.75 for Factor 3 (*health care*). For the older adult group, we found excellent reliability for the first factor (*health care* and *health promotion*), with Cronbach’s alpha at 0.92, and good reliability for the second factor (*disease prevention*), with Cronbach’s alpha at 0.78. Thus, we can conclude that the modified three-factor questionnaire in the adult group and the two-factor questionnaire in the older adult group exhibit good reliability in both age groups.

### 3.2 Discriminant validity

Discriminant validity of the HLS-EU-Q16 questionnaire was examined by its correlation with the communicative health literacy and navigational health literacy questionnaires. In the adult population (Table 6), the first dimension of the HLS-EU-Q16 questionnaire demonstrated good discriminant validity with the communicative health literacy questionnaire (COM-HL) (*r* = 0.47, *p* < 0.01) and the navigational health literacy questionnaire (NAV-HL) (*r* = 0.45, *p* < 0.01). The second dimension showed poorer discriminant validity with the COM-HL questionnaire (*r* = 0.19, *p* < 0.01) but good discriminant validity with the NAV-HL questionnaire (*r* = 0.48, *p* < 0.01). The third dimension also demonstrated good discriminant validity, with a correlation coefficient greater than 0.40 for both the COM-HL and NAV-HL questionnaires.

**Table 6:** Discriminant validity of the HLS-EU-Q16 in the adult population.

	COM-HL	NAV-HL
1. HLS-EU-Q16 (HP)	0.47**	0.45**
2. HLS-EU-Q16 (DP)	0.19**	0.48**
3. HLS-EU-Q16 (HC)	0.51**	0.57**

COM-HL, Communication Health Literacy Questionnaire; NAV-HL, Navigational Health Literacy Questionnaire; HP, Health promotion; DP, Disease prevention; HC, Health care. **p < 0.01.

For the older adult population (Table 7), the first dimension of the questionnaire showed excellent discriminant validity with the COM-HL (*r* = 0.62, *p* < 0.01) and NAV-HL (*r* = 0.66, *p* < 0.01). The second dimension showed adequate discriminant validity with the COM-HL questionnaire, as the correlation was slightly below the 0.40 threshold (*r* = 0.37, *p* < 0.01), and good validity with the NAV-HL (*r* = 0.61, *p* < 0.01). Overall, the discriminant validity of the HLS-EU-19 and its dimensions was confirmed in both age groups.

**Table 7:** Discriminant validity of the HLS-EU-Q16 in the older adult population.

	COM-HL	NAV-HL
1. HLS-EU-Q16 (HC, HP)	0.62**	0.66**
2. HLS-EU-Q16 (DP)	0.37**	0.61**

COM-HL, Communication Health Literacy Questionnaire; NAV-HL, Navigational Health Literacy Questionnaire; HP, Health promotion; DP, Disease prevention; HC, Health care. **p < 0.01.

### 3.3 Concurrent-predictive validity

Concurrent-predictive validity was assessed through linear regression analysis (see Tables 8, 9), where gender, age, education, self-assessed economic deprivation, and self-assessed socioeconomic status were included as predictors. In the adult population (Table 8), all variables were statistically significant predictors of the third dimension of the questionnaire, i.e., *health care*. In the first dimension, only age was not a statistically significant predictor, and in the second dimension, both gender and age did not significantly predict health literacy. All significant predictors of health literacy dimensions were in the expected direction, except for education, which negatively predicted the *disease prevention* dimension (*β* = −0.06, *p* < 0.01). Among older adults (see Table 9), socioeconomic status did not statistically significantly predict any dimension of the HLS-EU-Q16. Gender did not predict the first dimension, and education did not predict the second dimension. Significant predictors were in the expected direction. Since the majority of determinants were significant predictors of the questionnaire dimensions in both groups, we can conclude that the questionnaire has adequate concurrent-predictive validity.

**Table 8:** Concurrent-predictive validity in the adult population.

	HLS-EU-Q16 (HP)		HLS-EU-Q16 (DP)		HLS-EU-Q16 (HC)	
	*β* (S.E.)	*p*	*β* (S.E.)	*p*	*β* (S.E.)	*p*
Gender	0.13 (0.04)	< 0.001	0.07 (0.04)	0.092	0.08 (0.04)	< 0.001
Age	−0.04 (0.00)	0.070	−0.03 (0.00)	0.262	−0.04 (0.00)	0.044
Education	0.18 (0.01)	< 0.001	−0.06 (0.01)	0. 010	0.05 (0.01)	0.035
Economic deprivation	−0.13 (0.04)	< 0.001	−0.11 (0.04)	< 0.001	−0.19 (0.04)	< 0.001
Socioeconomic status	0.08 (0.02)	0.002	0.05 (0.02)	0.044	0.06 (0.02)	0.017

*R2 first dimension = 0.12. R2 second dimension = 0.02. R2 third dimension = 0.07. Shown are standardized beta coefficients. S.E., standard error; HP, Health promotion; DP, Disease prevention; HC, Health care.*

**Table 9:** Concurrent-predictive validity in the older adult population.

	HLS-EU-Q16 (HC, HP)		HLS-EU-Q16 (DP)	
	*β* (S.E.)	*p*	*β* (S.E.)	*p*
Gender	0.06 (0.07)	0.090	0.08 (0.07)	0.028
Age	−0.27 (0.01)	< 0.001	−0.22 (0.01)	< 0.001
Education	0.21 (0.02)	< 0.001	0.02 (0.02)	0.597
Economic deprivation	−0.19 (0.06)	< 0.001	−0.19 (0.06)	< 0.001
Socioeconomic status	0.04 (0.03)	0.407	0.07 (0.03)	0.128

*R2 first dimension = 0.19. R2 second dimension = 0.10. Shown are standardized beta coefficients. S.E., standard error; HP, Health promotion; DP, Disease prevention; HC, Health care.*

### 3.4 Concurrent validity

We assessed concurrent validity by examining the correlation coefficient between the three dimensions of the HLS-EU-Q16 in the adult population and two dimensions in the older adult population with the longer, original version of the questionnaire, HLS-EU-Q47. In the adult population we found that the concurrent validity of the questionnaire was excellent for the first (*r* = 0.81, *p* < 0.01) and third (0.79, *p* < 0.01) dimension, and satisfactory for the second dimension (*r* = 0.59, *p* < 0.01). For the older adults we found excellent concurrent validity for the first (*r* = 0.94, *p* < 0.01) and second (*r* = 0.76, *p* < 0.01) dimension. The high correlations thus indicate that the shorter HLS-EU-Q16 effectively measures the same construct of health literacy as the longer HLS-EU-Q47 among adults and older adults.

### 3.5 Predictive validity

We further performed hierarchical regression analyses regarding the predictive value of the HLS-EU-Q16 in predicting various health outcomes in both age groups. Among the adult sample (Table 10), we found that all three dimensions of the questionnaire statistically significantly predicted self-rated health so that higher levels of health literacy predicted better self-rated health. The inclusion of the three dimensions in the model for the self-rated health also statistically significantly increased the explained variance of the model (Δ*R*^2^ = 0.015, Δ*F* = 13.802, *p* < 0.001). For smoking, alcohol consumption, physical activity and healthy eating the three dimensions of the HLS-EU-Q16 were not able to explain significant variance over and above gender, age, education, economic deprivation and socioeconomic status. We did, however, find that the third dimension of the questionnaire—“*health care”*—significantly predicted smoking (*β* = 0.08, *p* < 0.01) but in an unexpected direction; higher health literacy was associated with more frequent smoking. Additionally, we found that the *“disease prevention”* dimension positively predicted physical activity (*β* = 0.05, *p* < 0.05), and *“health prevention”* positively predicted healthy eating (*β* = 0.05, *p* < 0.05). To summarize, the predictive validity of the questionnaire among adults was somewhat inadequate, as it mostly did not significantly increase the variance of the examined outcomes, and the dimensions were generally not significant predictors of health behaviors.

**Table 10:** Predictive validity of the HLS-EU-Q16 in the adult population.

	Self-rated health		Smoking		Alcohol consumption		Physical activity		Healthy eating	
	Step 1	Step 2	Step 1	Step 2	Step 1	Step 2	Step 1	Step 2	Step 1	Step 2
Gender	0.03	0.04*	−0.06**	−0.06**	−0.29***	−0.29***	−0.05*	−0.05*	0.15***	0.14***
Age	0.28***	0.27***	−0.09***	−0.09***	0.02	0.02	0.05*	0.05*	0.16***	0.16***
Education	−0.07*	−0.05*	−0.18***	−0.18***	0.05*	0.05*	−0.06*	−0.06*	−0.00	−0.01
ED	−0.11***	0.16***	0.07**	0.08**	−0.01	−0.02	−0.03	−0.02	−0.07**	−0.06*
SES		−0.10***	0.01	0.01	0.00	0.01	0.02	0.02	0.09***	0.08**
HLS-EU-Q16 (HP)		−0.07**		−0.04		−0.01		0.02		0.05*
HLS-EU-Q16 (DP)		−0.04*		−0.03		−0.01		0.05*		0.03
HLS-EU-Q16 (HC)		−0.06**		0.08**		−0.03		−0.00		−0.02
*R* ^2^	0.198	0.212	0.046	0.049	0.083	0.083	0.007	0.009	0.059	0.061
*F*	107.779***	73.735***	21.893***	14.873***	40.199***	25.438***	3.848**	3.309***	28.164***	18.553***
*ΔR* ^2^		0.015		0.004		0.001		0.003		0.003
*ΔF*		13.802***		3.068		0.849		2.398		2.441

ED, Economic deprivation; SES, Socioeconomic status; HP, Health promotion; DP, Disease prevention; HC, Health care. *p < 0.05, **p < 0.01, ***p < 0.001.

For the older adult sample (Table 11), we found that both dimensions of the HLS-EU-Q16 predicted better self-rated health and improved the explained variance of the regression model (Δ*R*^2^ = 0.015, Δ*F* = 13.802, *p* < 0.001). For the rest of the health behaviors, the inclusion of the health literacy questionnaire did not statistically significantly improve the explained variance of the outcomes. The analysis did confirm that disease prevention statistically significantly predicted more frequent physical activity (*β* = 0.05, *p* < 0.05), while the first dimension of the questionnaire—*health care and health promotion*—predicted healthy eating (*β* = 0.05, *p* < 0.05). Similar to the findings for the adult population, the HLS-EU-Q16 revealed unsatisfactory predictive validity, as it mostly failed to predict health behaviors among older adults.

**Table 11:** Predictive validity of the HLS-EU-Q16 in the older adult population.

	Self-rated health		Smoking		Alcohol consumption		Physical activity		Healthy eating	
	Step 1	Step 2	Step 1	Step 2	Step 1	Step 2	Step 1	Step 2	Step 1	Step 2
Gender	−0.00	0.01	−0.10**	−0.10**	−0.34***	−0.33***	0.01	−0.00	0.15***	0.14***
Age	0.20***	0.14***	−0.21***	−0.20***	−0.04	−0.06	−0.22***	−0.17***	−0.08*	−0.07
Education	−0.09*	−0.04	−0.02	−0.03	0.05	0.06	0.00	−0.03	−0.02	−0.04
ED	0.21***	0.17***	0.03	0.03	−0.06	−0.08	−0.05	−0.01	−0.07	−0.06
SES	−0.09*	−0.08	−0.05	−0.05	0.01	0.01	0.03	0.03	0.07	0.07
HLS-EU-Q16 (HC. HP)		−0.24***		0.03		−0.06		0.14**		0.11*
HLS-EU-Q16 (DP)		0.02		−0.03		−0.03		0.06		−0.05
*R* ^2^	0.134	0.176	0.051	0.049	0.129	0.131	0.048	0.069	0.031	0.036
*F*	24.517***	24.054***	9.048***	6.548***	23.211***	17.234***	8.625***	9.042***	5.826***	4.961***
*ΔR* ^2^		0.043		0.001		0.005		0.024		0.007
*ΔF*		19.827***		0.340		2.119		9.590***		2.731

ED, Economic deprivation; SES, Socioeconomic status; HP, Health promotion; DP, Disease prevention; HC, Health care. *p < 0.05, **p < 0.01, ***p < 0.001.

## 4 Discussion

Health literacy is considered an important determinant of health and health behavior (34–36). Thus, it is critical to use psychometrically appropriate tools to accurately measure the health literacy of individuals. The HLS-EU questionnaires are widely used for assessing health literacy. Therefore, this study aimed to evaluate the validity and reliability of the HLS-EU-Q16 questionnaire among a Slovenian sample of adults and older adults. Examining the validity and reliability of health literacy questionnaires in different age groups, including target groups such as the older adult who tend to have lower levels of health literacy, is also important for understanding and addressing their specific health literacy needs.

First, construct validity was assessed using confirmatory factor analysis (CFA) based on the theoretical structure of three factors: health care, disease prevention, and health promotion (2). The analysis indicated poor model fit in both age groups. Consequently, we re-examined the factor structure using exploratory factor analysis (EFA), which revealed different structures for each group. Among adults, a three-factor structure emerged, with some overlap between health promotion, disease prevention, and health care. This finding suggests that adults may perceive health promotion in a broader context. For example, adults may interpret activities such as managing well-being or understanding advice from family members as part of health promotion, which indicates that health literacy in this population extends beyond traditional categories into a more fluid perception of health. In contrast, a two-factor structure was identified in the older adult population, demonstrating a more holistic approach to health literacy. Older adults seem to perceive health care and health promotion as interconnected. This consolidation suggests that older adults might prioritize practical health management over distinguishing between health promotion, disease prevention, and health care. Instead, they may see these aspects as part of a cohesive strategy to maintain health and well-being. Additionally, older adults grouped items related to disease prevention—such as media reliability and protective measures—into a single factor, potentially reflecting their greater reliance on external information regarding health. Therefore, these findings highlight the dynamic nature of health literacy across different life stages. For adults, health literacy involves a broad understanding of how to promote health and prevent disease, suggesting that interventions targeting this group may need to address these interconnected aspects more explicitly. For older adults, however, the emphasis on a more integrated approach to health management indicates that health literacy interventions should focus on helping them navigate the complex interactions between healthcare services, disease prevention, and health promotion in a way that resonates with their more holistic view of health.

Our findings suggest that construct validity of the HLS-EU-Q16 questionnaire in Slovenia is appropriate for both adults (the modified three-factor structure) and older adults (two-factor structure). Although some previous studies confirmed the theoretically proposed three-factor solution (5, 12, 21), it is not surprising that different structures emerged in our study. The originally proposed three-structure of the questionnaire has not been consistently supported in prior research, with some studies identifying four-factor solutions (9, 13). In addition, contrary to findings supporting a one-factor (higher order) structure of the questionnaire (14, 23), we did not find such evidence, as model fit statistics fell below acceptable thresholds in both groups. This suggests that the HLS-EU-Q16 should not be examined as a single factor in future research in Slovenia.

Next, discriminant validity was confirmed in both age groups, indicating that the constructs measure distinct aspects of health literacy without multicollinearity, consistent with Pelikan et al. (3) findings for the HLS_19_-Q12 version of the questionnaire. Regarding concurrent predictive validity, most sociodemographic determinants significantly predicted health literacy. For example, in the adult sample, we found that age and socioeconomic status statistically significantly predicted all three HLS-EU-Q16 dimensions, aligning with findings from a study on Hungarian mothers (48).

Finally, with regard to predictive validity, several previous studies have demonstrated a positive relationship between health literacy and self-rated health (34, 38). Our findings align with these results, as all three dimensions of the HLS-EU-Q16 significantly predicted self-rated health among adults. However, our study found limited predictive validity for other health behaviors such as smoking, alcohol consumption, physical activity, and healthy eating. This is in line with the results by Aaby et al. (49) who examined health literacy using two subscales of the HLQ questionnaire: understanding health information and the ability to actively engage with healthcare providers. They found that while health literacy, particularly understanding health information, does predict several health behaviors, its effect is inconsistent for alcohol consumption, dietary quality score, and certain BMI categories. The ability to actively engage with healthcare providers does not consistently predict former alcohol consumption, underweight status, average dietary quality, or obesity when adjusting for other factors (41). Similarly, a study by Tiller et al. (50) examined the HLS-EU-Q16 as a predictor of health outcomes among older adults and found that health literacy did not significantly predict blood pressure. This suggests that the relationship between health literacy and certain health behaviors is complex and might be influenced by other factors.

One potential explanation for the limited predictive validity of health behaviors in our study could be the role of mediating factors. Findings from a meta-analysis support this notion, revealing that the relationship between health literacy and health behaviors is mediated by other determinants like self-efficacy (51). Individuals with higher health literacy may not automatically engage in healthy behaviors unless they also feel confident in their ability to act. For example, someone with adequate health literacy might understand the risks of smoking or the benefits of healthy eating, but if they lack self-efficacy, they may not translate this knowledge into action. Moreover, Wieczorek et al. (52) found that social connectedness moderates the relationship between health literacy and health behaviors, further complicating direct predictions. Social networks and support systems can influence the ability to engage in healthy behaviors, particularly for older adults who may rely more on family and community for health-related decisions. We should note that we did not take social connectedness nor self-efficacy into account in our study as we lacked measures. For older adults, the HLS-EU-Q16 also showed limited predictive validity, similar to the adult sample. While it significantly predicted self-rated health, it did not substantially improve the explained variance for other health behaviors. This means that other factors may play a potential role in the relationship between health literacy and health behaviors.

To summarize, HLS-EU-Q16 showed reliability and validity in Slovenian adults (modified-three factor structure) and older adults (two-factor structure), suggesting it can be used in future research or in practical settings. This is particularly advantageous as researchers can adopt the shorter version instead of the longer HLS-EU-Q47, lowering the burden on respondents and allowing them to fill out the questionnaire faster. This also reduces the cost of studies. Healthcare professionals can utilize the HLS-EU-Q16 to assess patients’ health literacy levels, enabling them to tailor their communication and healthcare instructions accordingly. Despite demonstrating limited predictive validity for predicting certain health behaviors, the tool’s ability to reliably predict self-rated health highlights its value in clinical settings.

### 4.1 Limitations and future research

The present study focused on the validity of the HLS-EU-Q16 questionnaire among adults and older adults in Slovenia, which may limit the generalizability of the results to other national contexts. We did not examine specifics regarding national identity or gender. The data were self-reported, which could lead to socially desirable answers or inaccurate representations of behaviors. Additionally, the cross-sectional design of the study prevents us from making causal inferences.

Since we did not confirm the proposed three-factor structure of the questionnaire in both age groups, as different factor structures emerged in both groups, we suggest that future studies explore the construct validity on their samples before using the construct for further analyses to ensure the accuracy of the results. The findings also suggest that while health literacy is a critical factor in self-rated health, its role in predicting specific health behaviors may be limited and mediated by other factors, which should be further investigated. Future studies should also examine the predictive validity of the questionnaire concerning other health behaviors and attitudes, such as vaccination uptake.

## 5 Conclusion

The HLS-EU-Q16 was shown to be a reliable and valid tool for assessing health literacy among Slovenian adults and older adults. This study’s findings support its use in both research and clinical settings, offering a practical alternative to the longer HLS-EU-Q47. The distinct factor structures identified for different age groups suggest that health literacy is perceived variably across the lifespan, necessitating age-specific approaches. While the questionnaire demonstrated strong predictive validity for self-rated health, its limited predictive validity for other health behaviors highlights the complex interplay of factors that influence health literacy outcomes. Consequently, health literacy interventions should address additional determinants such as education, economic status, and access to healthcare resources to be more effective.

## Data Availability

The dataset used in this study is not yet publicly available due to NIJZ’s research project restrictions. Requests to access these datasets should be directed to National Institute of Public Health Slovenia: https://nijz.si/.
