# Supplementary material for: The validity and reliability of the Slovenian version of the health literacy questionnaire short-form (HLS-EU-Q16) among adults and older adults
Source: Front Public Health. 2024 Dec 11;12:1474539. doi: 10.3389/fpubh.2024.1474539 (PMC11670326; doi:10.3389/fpubh.2024.1474539)
Supplement: Supplementary file 1 [file Data_Sheet_1.docx]

Supplementary Material

Table S1. *Construct Validity of the HLS-16 Questionnaire with theoretically proposed dimensions*

|  | Adults | Older adults |
| --- | --- | --- |
| χ² (df) | 1791,552 (101)*** | 725,601 (101)*** |
| CFI | 0,88 | 0,91 |
| TLI | 0,86 | 0,90 |
| RMSEA | 0,09 | 0,08 |
| SRMR | 0,05 | 0,05 |

*Note.* CFI = Comparative Fit Index, TLI = Tucker-Lewis Index, RMSEA = Root Mean Square Error of Approximation, SRMR = Standardized Root Mean Square Residual. *** *p* < 0.001.

Table S2. *Construct validity of the one-factor HLS-EU-Q16 Questionnaire*

|  | Adult population | Older adult population |
| --- | --- | --- |
| χ² (*df*) | 1634.412 (104)*** | 746.908 (104)*** |
| CFI | 0.85 | 0.88 |
| TLI | 0.83 | 0.86 |
| RMSEA | 0.08 | 0.08 |
| SRMR | 0.06 | 0.05 |

*Note.* CFI = Comparative Fit Index, TLI = Tucker-Lewis Index, RMSEA = Root Mean Square Error of Approximation, SRMR = Standardized Root Mean Square Residual. *** *p* < 0.001.
